# Socioeconomic Position Across the Life Course and Trends in Disability-Free Life Expectancy in Australia

**DOI:** 10.1093/geroni/igaa057.2199

**Published:** 2020-12-16

**Authors:** Kim Kiely, Richard Tawiah, Carol Jagger, Kaarin Anstey

**Affiliations:** 1 University of New South Wales, Sydney, New South Wales, Australia; 2 University of New South Wales, Bankstown, New South Wales, Australia; 3 Newcastle University, Newcastle upon Tyne, England, United Kingdom; 4 University of New South Wales, Sydney, Australia

## Abstract

There has been little investigation of how life-course social mobility is linked to Disability-Free Life Expectancy (DFLE). We report novel analysis of the HILDA survey examining how DFLE trends differs by three markers of socio-economic position (SEP): early-life (educational attainment), midlife (occupational level), and late-life (area-disadvantage). All women, irrespective of their educational level, gained years with disability (Age 65: Low education=1.5 and High education=2.5 years). Similar results were obtained by level of occupation, but women with low occupation showed small declines in LE (-0.8 years), all being losses in DFLE. Only women in the most advantaged areas gained DFLE. For men, higher levels of any marker of SEP were associated with DFLE gains that were larger than, or comparable to, gains in years lived with disability, although lower education was associated only with gains in years lived with disability. DFLE trends differ by SEP marker more in women than men.

